# Relationship Between Perceived Organizational Support, Work Well-Being, and Medical Narrative Ability Among Nurses: A Cross-Sectional Multicenter Study

**DOI:** 10.1155/jonm/4466721

**Published:** 2024-12-17

**Authors:** Yanjia Li, Limei Kang, Rong Zhang, Yanli Hu, Limei Zhang, Xiaoying Zeng, Fengju Wu, Xiao He, Yiying Zhang, Jing Liu, Shurong Tang

**Affiliations:** ^1^Department of Nursing, Sichuan Taikang Hospital, Chengdu 610213, Sichuan, China; ^2^School of Nursing, Guangzhou Medical University, Guangzhou 511495, Guangdong, China; ^3^School of Nursing, Chengdu Medical College, Chengdu 610500, Sichuan, China; ^4^Neurology Department, Ziyang Central Hospital, Ziyang 641300, Sichuan, China

## Abstract

**Aims:** The purpose of this study was to investigate the relationship between nurses' perceived organizational support, work well-being, and medical narrative ability.

**Background:** With the proposed bio-psycho-social medical model, nurses' medical narrative ability is closely related to patients' health problems and quality of life. Nurses' perceived organizational support and work well-being can improve nurses' empathy and reflection ability to a certain extent and promote patients' rehabilitation. However, the relationship between nurses' perceived organizational support, work well-being, and medical narrative ability is unclear.

**Methods:** A total of 1831 nurses from 8 hospitals in China were surveyed using an online questionnaire that included nurses' sociodemographic, perceived organizational support, work well-being, and medical narrative ability. IBM SPSS 27.0 was used for Pearson's correlation analysis, one-way ANOVA, *t*-test, and mediation effect analysis using Model 4 in PROCESS (5000 resamples).

**Results:** The total mean score of perceived organizational support (46.68 ± 11.00), work well-being (53.09 ± 10.81), and medical narrative ability (154.48 ± 22.93) among nurses was found to be moderate. The relationship between perceived organizational support, work well-being, and medical narrative ability was significant, with correlation coefficients ranging from 0.348 to 0.685 (*p* < 0.01). The relationship between perceived organizational support and medical narrative ability is partially mediated by work well-being. The intermediate effect accounted for 52.36% of the total effect.

**Conclusion:** This study found that nurses' work well-being mediated the relationship between perceived organizational support and medical narrative ability.

**Implications for Nursing Management:** This study evaluated nurses' medical narrative ability and explored the relationship between nurses' perceived organizational support, work well-being, and medical narrative ability. The results of this study can help nursing managers and educators to take appropriate measures to intervene nurses' perceived organizational support and work well-being, so as to improve nurses' medical narrative ability and optimize nursing quality.

## 1. Introduction

With the proposed bio-psycho-social medical model, the prevention and treatment of diseases should pay attention to not only biochemical factors but also social and psychological factors [1]. Therefore, the meaning of health is gradually being enriched. In 1998, the WHO proposed that health is a dynamic state of complete physical, mental, spiritual, and social well-being and not merely the absence of disease or infirmity [2]. Although the new health connotation has pointed out the importance of psychological factors to disease and health, in some medical institutions, nurses pay more attention to patient's symptoms, and their ability to sympathize with patients and perceive their plight seems lacking [3]. However, people's requirements for the quality of medical services are gradually increasing. Due to the lack of effective communication between nurses and patients, nurses do not pay attention to the emotional experience of patients, which results in a tense nurse–patient relationship and an increasing number of violent injuries to medical staff [4]. How to effectively help nurses better deal with patients' emotional responses during disease treatment to achieve a real sense of humanistic care has become an urgent problem for society to solve. Ana et al. [5] showed that nurses with high medical narrative ability could reduce nurse–patient conflicts and establish a harmonious and stable nurse–patient relationship.

The concept of medical narrative ability was first proposed by Rita Charon [6] in 2001, which refers to “the ability to absorb, explain and respond to stories and other people's difficulties.” Its core competence is reflection and empathy, which refers to the ability of medical staff to recognize and understand the patient's disease story, use their narrative ability to accurately explain the deep meaning behind the story, be moved and empathized by it, and act for the benefit of the patient. The concept of “medical narrative ability” is proposed in the context of patient-centered medicine, and it is distinguished from “patient-centered care” or “clinical empathy.” Patient-centered nursing is a modern medical concept that emphasizes respecting patients' wishes and needs, paying attention to their individual differences, and providing them with comprehensive, continuous, and effective nursing services [7]. However, medical narrative ability focuses more on the reflection and empathy abilities of medical staff. Its purpose is to build a good relationship between doctors and patients, enhance the professional value of medical staff, and promote the development of humanistic nursing or humanistic medicine [8]. It is an integral part of patient-centered nursing [8]. In addition, clinical empathy refers to the ability to empathize with and understand others' feelings and perspectives [9]. It has become a necessary skill for medical staff as it can improve patient satisfaction and treatment outcomes. Furthermore, it is one of the core abilities encompassed by medical narrative ability as it can impact individual's medical narrative ability [10]. Feng et al. [11] showed that nurses with medical narrative ability can actively listen to patients' narratives, deepen nurses' understanding of patients' diseases, meet patients' psychological and spiritual needs, alleviate patients' loneliness and pain, and promote patients' recovery from diseases. However, narrative medicine was introduced in China in 2011, and domestic research on nurses' medical narrative ability is still in the primary stage. According to the literature review, nurses' level of medical narrative ability in China is generally low [8], which is affected by nurses' work experience, narrative medicine training experience, work pressure, social support, and other factors [12]. More importantly, nurses' medical narrative ability is closely related to patient's health problems and quality of life [13]. In response to these concerns, how to improve nurses' medical narrative skills has become a vital issue in the healthcare industry.

Perceived organizational support refers to employees' subjective feelings about how the organization views their contributions and cares about their interests [14]. Based on the principle of benefit reciprocity in social exchange theory, when one party gives support and help to the other party, the other party should return [15]. Zaid et al. [16] showed that perceived organizational support can stimulate nurses' work enthusiasm, enhance their sense of belonging to the organization, make them feel the care and help from the organization, actively return work to the organization, improve nurses' professional identity, provide patients with better nursing services, and improve patient satisfaction. In addition, the study of Dan et al. [17] shows that perceived organizational support is conducive to building a good nurse–patient relationship and medical care relationship, creating a warm department atmosphere, making nurses feel safe and trusted, alleviating job burnout [18], improving their enthusiasm for work, and enhancing their willingness to learn narrative nursing [19]. However, it is still being determined whether nurses' perceived organizational support contributes to improving nurses' medical narrative ability.

With the rise of positive psychology, work well-being is defined as the positive evaluation derived from an individual's perception of the whole work process, including the physical work environment and subjective emotional evaluation [20]. The research of Rebecca et al. [21] shows that nurses face a high-pressure working environment for a long time and have heavy nursing tasks, so their work well-being is lower than that of other people. Emily et al. [22] believed that a positive coping style could enhance nurses' work well-being and job satisfaction, relieve nurses' work pressure and psychological pain, and enhance nurses' empathy and reflective ability. Empathy and reflective ability are the core elements of nurses' medical narrative ability [23], and research shows that nurses' job satisfaction can improve their medical narrative ability [24]. Therefore, we hypothesized a link between nurses' work well-being and their medical narrative ability.

The relationship between nurses' perceived organizational support, work well-being, and medical narrative ability can be explained by the job demand–resource model combined with positive psychology. According to the demand–resource model [25], perceived organizational support is a crucial work resource for nurses. It can stimulate their work initiative, help nurses effectively deal with various setbacks in work, and improve their work self-confidence and subjective well-being. In addition, positive psychology believes that positive emotions have an important impact on people's physical and mental health, happiness, and personal ability (professional ability, empathy ability, learning ability) [26]. These findings provide valuable theoretical and practical implications for implementing interventions to improve caregivers' medical narrative ability. When nurses have a high level of perceived organizational support, they can devote themselves to their work, stimulate their work enthusiasm and work well-being [27], realize their own life value and significance, and enhance their sense of career benefit and empathy [28]. They will be able to think from the perspective of the patient, listen to the story behind the patient's illness, and pay attention to the patient from the psychological and spiritual level, thus improving the nurse's medical narrative ability. Therefore, we hypothesize that there is a positive correlation between perceived organizational support, work well-being, and medical narrative ability and that work well-being plays an intermediary role between perceived organizational support and medical narrative ability.

With the increasing population aging, the world faces a shortage of nurses. Surprisingly, few empirical studies have investigated the relationship between nurses' sense of perceived organizational support, work well-being, and medical narrative ability, especially in China. Therefore, in this study, we aim to explore the relationship between nurses' perceived organizational support, work well-being, and medical narrative ability and whether nurses' work well-being plays an intermediary role in the relationship between the perceived organizational support and medical narrative ability. Our findings can help nursing administrators and educators implement effective interventions to improve nurses' medical narrative ability, build a harmonious nurse–patient relationship, improve clinical nursing quality, promote patients' early recovery, improve patient satisfaction, and promote the development of humanistic nursing. Based on the above content, this study proposes the following research hypotheses:

• Hypothesis 1. Perceived organizational support is positively correlated with medical narrative ability.
• Hypothesis 2. Work well-being is positively correlated with medical narrative ability.
• Hypothesis 3. Perceived organizational support is positively correlated with work well-being.
• Hypothesis 4. Work well-being plays a mediating role between perceived organizational support and medical narrative ability.

## 2. Methods

### 2.1. Design, Setting, and Participants

A multicenter cross-sectional questionnaire design was adopted to facilitate sampling, and 1831 nurses from 8 hospitals across the country were surveyed in January–February 2024. The inclusion criteria were as follows: (i) age ≥ 18 years old; (ii) obtain the professional qualification registration of nurses; (iii) voluntary participation in this study. The exclusion criteria are as follows: (i) nurses who were not on duty during the survey period; (ii) withdrawal of researchers. Accordingly, to fulfill the SEM analysis requirements and enhance the reliability and validity of our findings, we need to gather at least 204 completed and valid questionnaires [29]. These questionnaires provided demographic information and captured details about the nurses' medical narrative abilities, work well-being, and perceived organizational support.

### 2.2. Variables and Instruments

The questionnaire included sociodemographic characteristics (gender, age, marital status, education level, department, working experience, income, receive narrative medicine training, and participate in social activities every week), the medical narrative ability, work well-being, and the perceived organizational support.

Nurses' medical narrative ability was measured using the Medical Narrative Ability Scale, which was developed by Wanzhen et al. [30]. The Medical Narrative Ability Scale has 27 items: pay attention and listen (9 items), understanding and response (12 items), and reflection and representation (6 items). The items are rated on a 7-point Likert scale ranging from 1 (*completely inconsistent*) to 7 (*completely consistent*). In order to better present the results, the original authors of the Medical Narrative Ability Scale divided their results into three levels based on the total score of the scale: low (27–144), medium (145–163), and high (164–189). Cronbach's alpha coefficient of the Medical Narrative Ability Scale was 0.967 in this study.

Nurses' work well-being was measured using the Work Well-Being Scale, which was developed by Yajing [31]. The Work Well-Being Scale has 15 items: positive emotion (4 items), negative emotion (5 items), and work satisfaction (6 items). The items are rated on a 5-point Likert scale ranging from 1 (*completely inconsistent*) to 5 (*completely consistent*). In order to better present the results, the original authors of the Work Well-Being Scale divided their results into three levels based on the total score of the scale: low (15–31), medium (32–58), and high (59–75). Cronbach's alpha coefficient of the Work Well-Being Scale was 0.929 in this study.

Nurses' perceived organizational support was measured using the Perceived Organizational Support Scale, which was developed by Zhixia et al. [32]. The Perceived Organizational Support Scale has 13 items: emotional support (10 items) and instrumental support (3 items). The items are rated on a 5-point Likert scale ranging from 1 (*completely inconsistent*) to 5 (*completely consistent*). In order to better present the results, the original authors of the Perceived Organizational Support Scale divided their results into three levels based on the total score of the scale: low (13–27), medium (28–50), and high (51–65). Cronbach's alpha coefficient of the Perceived Organizational Support Scale was 0.981 in this study.

### 2.3. Data Collection

We randomly selected 8 hospitals in China by convenient sampling, collected questionnaires in each hospital with the help of members of a professional academic organization (Health Narrative Professional Committee), and conducted training for researchers before the investigation. Before the investigation, the researchers screened the survey subjects according to the inclusion and exclusion criteria strictly, and then sent a survey notice through social communication programs (such as FeiShu, WeChat, QQ) to inform the surveyed nurses of the purpose and significance of the study, as well as the notes for answering and the time to fill in the questionnaire. After obtaining the consent of the respondents, the researchers sent links to online questionnaires for data collection. In order to ensure the accuracy and validity of the data, after the participants completed the questionnaire, two investigators jointly checked whether the questionnaire was complete. According to the data results of the pre-survey and the questionnaire filling instructions of the original author, if the questionnaire filling time in this study is less than 5 min or more than 30 min, the survey results are invalid.

### 2.4. Ethical Consideration

This study was approved by the Ethics Committee of Sichuan Taikang Hospital (No. SCTK-IRB-2024-014). The questionnaire was filled in anonymously, the informed consent of the respondents was strictly observed before filling in the questionnaire, and the respondents could choose to withdraw from the study at any time. In addition, we ensure that the personal information of each participant is kept confidential, and the results of the questionnaire are only used for statistical analysis.

### 2.5. Data Statistics

Data were analyzed using IBM SPSS 27.0. Normal distributions for nurses' medical narrative abilities, work well-being, and perceived organizational support were examined using quantile–quantile plotting. Descriptive analysis of all data was performed using percentages, averages, and standard deviations. The relationship between nurses' medical narrative ability, perceived organizational support, and work well-being was compared by Pearson correlation analysis. The analysis of one-way ANOVA and *t*-test were used to compare the demographic differences in nurses' medical narrative ability. Harman's single-factor test was used to examine common method variance with self-report measures. Model 4 of Hayes' PROCESS macro in IBM SPSS 27.0 was used to perform bootstrapping to test the mediating role of work well-being in the relationship between perceived organizational support and medical narrative ability. The bootstrap method with a 95% bias-corrected confidence interval (CI) was used to test the significance of the mediation effect. The statistical significance was set at *p* < 0.05 with two-tailed testing.

## 3. Results

In this study, over half participants were women (97.4%) and most participants (60.1%) were aged 26–35. In addition, 73.6% participants were married, 71.3% participants had a bachelor's degree or above, and 37.6% participants were nurses working in internal medicine. About half of the participants (53.4%) had a monthly income of 5001–10,000 RMB, 51.8% had work experience of 6–15 years, 49.6% of the participants had no narrative medicine training, and 58.7% participated in social activities every week. The one-way ANOVA and *t*-test showed significant differences in nurses' medical narrative ability in terms of age, marital status, department, working experience, receiving narrative medicine training, and participation in social activities every week (*p* < 0.05). The one-way ANOVA and *t*-test showed significant differences in nurses' perceived organizational support in terms of department and participation in social activities every week (*p* < 0.05). The one-way ANOVA and *t*-test showed significant differences in nurses' work well-being in terms of age, income, and working experience (*p* < 0.05) (Table 1).

### 3.1. Descriptive Statistics of Main Study Variables

In this study, the total mean score of nurses' medical narrative ability, work well-being, and perceived organizational support were 154.48 ± 22.93, 53.09 ± 10.8, and 46.68 ± 11.00, respectively. The specific dimension scores of each scale are shown in Table 2.

### 3.2. Correlation Among Major Variables

In this study, nurses' medical narrative ability was positively correlated with the work well-being (*r* = 0.391, *p* < 0.01) and the perceived organizational support (*r* = 0.348, *p* < 0.01). In addition, the work well-being is also positively correlated with the perceived organizational support (*r* = 0.685, *p* < 0.01). Detailed results are shown in Table 3, which initially support our research hypothesis.

### 3.3. Mediating Effect of Nurse's Work Well-Being Between Perceived Organizational Support and Medical Narrative Ability

**Figure 1:**
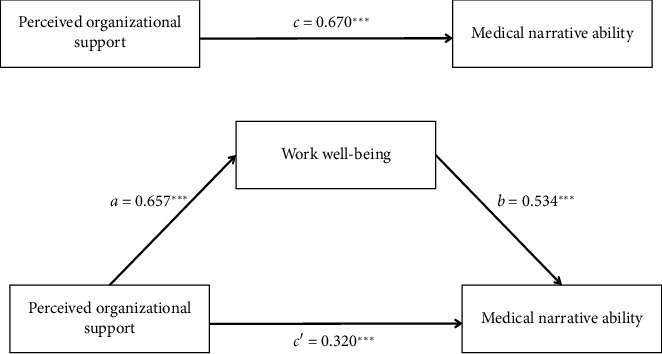
Mediation model of work well-being on the relationship between perceived organizational support and medical narrative ability of nurses. ⁣^∗∗∗^*p* < 0.001.

Harman's single-factor test showed that the cumulative variance interpretation was 33.91%, which is lower than the critical standard of 40% [33], illustrating that common method variance was not significant in this study. The study assessed the mediating role of work well-being on the relationship between perceived organizational support and medical narrative ability. Control variables were age, marital status, department, working experience, receiving narrative medicine training, and participation in social activities every week. The results showed that perceived organizational support significantly predicted work well-being (*a* = 0.657, SE = 0.017, *p* < 0.001), work well-being was shown to be a significant predictor of medical narrative ability (*b* = 0.534, SE = 0.064, *p* < 0.001), and perceived organizational support also had a direct effect on medical narrative ability (*c*′ = 0.320, SE = 0.061, *p* < 0.001). The bias-corrected percentile Bootstrap method test showed that 95% CI did not include 0, indicating that work well-being partially mediated the relationship between perceived organizational support and medical narrative ability. The mediation effect accounted for 52.36% of the total effect. The Mediation analysis summary is presented in Table 4 and Figure 1.

## 4. Discussion

This study investigated the status quo of nurses' medical narrative ability, perceived organizational support, and work well-being and explored the relationship between these three variables and the possible mediating role of work well-being in the relationship between perceived organizational support and medical narrative ability. First of all, in this study, nurses' medical narrative ability was at an average level, similar to the findings of Feng et al. [11]. In this study, the older the nurses were and the longer the working experience, the higher their medical narrative ability was, but the overall medical narrative ability was still not high. Compared with other countries, the introduction of narrative nursing in China is relatively late, and related research on narrative nursing is in its infancy [10], so the medical narrative ability of older nurses and longer working experience has not been significantly improved. In addition, nurses working in the outpatient department had the highest medical narrative ability, while nurses working in the internal department had the lowest medical narrative ability. It may be due to the fact that the outpatient department usually pays more attention to nursing etiquette and nurse–patient communication, and the large number of patients in the medical department, the high pressure of nurses' work, coupled with the lack of practical guidance and training of nurses related to narrative nursing, resulting in their low medical narrative ability [8]. In this study, 49.6% of nurses had not participated in relevant training on narrative nursing and had less understanding of narrative nursing and lacked opportunities for theoretical learning and practice [34]. At the same time, this study also shows that nurses who have participated in narrative medicine–related training have significantly higher medical narrative ability than nurses who have not participated in the training, which also indicates the importance of narrative medicine training. At present, some medical and educational institutions in China have not paid enough attention to the training of nurses' medical narrative ability, and medical colleges have not incorporated narrative medicine or narrative nursing–related courses into nursing personnel training programs, making the concept of narrative nursing not yet popular in clinical practice [35]. The results of this study also showed that nurses who regularly participated in social activities had higher medical narrative ability than those who did not participate in social activities. The study showed that necessary social activities could promote the physical and mental health of individuals, relieve work pressure, and coordinate interpersonal relationships, so they could better engage in medical work and promote the improvement of personal medical narrative ability [36]. Hong et al. [37] showed that nurses' medical narrative ability is crucial to patients' physical, psychological, social, and spiritual health. This suggests that nursing managers and educators should prioritize cultivating nurses' medical narrative ability.

In this study, the total average score of nurses' perceived organizational support was similar to Mingjing et al. [38] but lower than that of Mei et al. [39]. These findings suggest that nurses have moderate perceived organizational support. It may be related to the differences in the strength of encouragement and support for nurses in various hospitals and related policies, and it may also be related to the time of this survey. This study showed that nurses working in the pediatric department had the highest perceived organizational support and those working in the emergency department had the lowest perceived organizational support. This may be due to the difficulty of the job and the high turnover rate of pediatric nurses, so the training of pediatric nurses has been a global priority, so pediatric nurses feel the most organizational support [40]. The emergency department inevitably has more doctor–patient disputes, the working environment and work tasks of emergency nurses are also challenging, and some of the surveyed nurses in this study are nurses in private hospitals, who tend to receive less organizational support [41]. In addition, the nurses in this study who participated in social activities every week had a higher perceived organizational support. When the organization gave sufficient emotional and instrumental support, the stronger the individual's subjective well-being, the more time and energy the individual would have to participate in social activities [42]. However, the nurses' perceived organizational support in this study was at a moderate level, which may be mainly due to the huge impact of the COVID-19 epidemic that lasted several years on the entire healthcare system [43]. Some hospitals have taken measures such as cutting expenses, controlling costs, and reducing staff salaries to cope with risks such as capital turnover difficulties and reduced operational effects [44]. In such an organizational environment, it is difficult for nursing staff to obtain emotional and instrumental attention and support from the hospital. The International Council of Nurses (ICN) also noted that “we have seen time and time again that financial crises often lead to constraints on healthcare budgets, often at the expense of nursing services; despite being the backbone of health care, nursing often faces financial constraints and societal undervaluation.” The theme of International Nurses Day 2024 is “our nurses, our future, the economic power of care” [45], which suggests that healthcare institutions and medical school administrators can invest more in nursing, improve nurses' working conditions and safety, expand the workforce's size, improve nursing education, and enhance nurses' perceived organizational support.

In this study, nurses reported a moderate level of work well-being, similar to a recent study's findings [46]. Work well-being is a positive psychological experience generated by nurses at work, which will affect their work attitude and physical and mental health [47]. Nearly half of the nurses in the study worked in medical nursing. With the increasing aging of the population, there are more and more chronic disease patients in China, nurses are in short supply, work pressure is high, tasks are heavy, and risks are high. The imbalance between the work effort and income of nurses easily leads to conflicts between work and family, resulting in negative emotions. Secondly, the social recognition of nurses in China is low, and their professional identity is not strong, which makes it difficult to stimulate the work motivation of nurses [48]. Finally, the irregular work and rest time disturb the normal life of nurses. The tense work rhythm causes nurses to lack sufficient time to adjust their mental and physical health; damages their endocrine system, immune system, and mental system; and induces physical and mental diseases [49], which leads to an increase in negative emotions, makes nurses often show fatigue and job burnout, and reduces the work well-being. This study also showed that the older the nurse, the longer the working experience, and the higher the monthly income, the higher the work well-being. Therefore, nursing managers should pay attention to the career development of nurses with different ages, different working experience, and different incomes; increase the wage subsidy for nurses; and provide multichannel promotion opportunities, especially the nurses with low seniority and low income. They should also rationally allocate human resources and strengthen mental health guidance to nurses to improve their work well-being.

According to our research results, nurses' perceived organizational support was positively correlated with their work well-being, indicating that the higher the level of nurses' perceived organizational support, the stronger their work well-being. Studies have found that perceived organizational support can improve nurses' work enthusiasm, reduce negative emotions, and enhance nurses' job satisfaction and positive emotions [50]. When medical institutions create a supportive and trustworthy organizational environment, nurses' work passion can be stimulated, thus promoting their higher work well-being [51]. Second, our findings showed that nurses' perceived organizational support was positively correlated with medical narrative ability. When the organization provides sufficient emotional support and tool support to employees, it helps to create a warm working atmosphere, make nurses feel safe and trusted, relieve job burnout, improve their enthusiasm for work, and enhance their cohesion and empathy [52] to promote the improvement of their medical narrative ability. Finally, this study found that the higher the nurses' work well-being, the stronger their medical narrative ability. The study found that the higher the nurses' job satisfaction and sense of career benefit [53], the greater the impact on the nurses' work well-being. They are more willing to engage in work to communicate with patients, solve their problems, and constantly reflect on whether their work needs improvement. According to the theory of humanistic nursing care [54], nursing managers' care for nurses can directly affect the interaction between nurses and patients. When nurses feel a certain degree of care and happiness, they will be more willing to pass on warmth to people in need, forming a positive professional value and actively providing narrative help to patients other than treatment. Then, it improves nurses' medical narrative ability.

Finally, the most important finding in this study is that nurses' work well-being mediates between perceived organizational support and medical narrative ability. According to the demand–resource model of work [25], perceived organizational support is an important work resource for nurses, which can stimulate their work enthusiasm and inner positive psychological quality, calmly face difficulties in work, improve job satisfaction, and reduce negative emotions and job burnout. As a variable positive psychological resource with similar status [55], work well-being is studied as an intermediary variable in this study. It is an intrinsic motivating factor of nurses' medical narrative ability and positive emotional experience. It plays a key role in the relationship between nurses' perceived organizational support and medical narrative ability. When nurses get enough organizational support, it can stimulate their work well-being, promote their positive emotional experience [47], enhance their empathy and reflective ability, build a harmonious nurse–patient relationship, and further improve their medical narrative ability. In addition, when nurses receive less organizational support, they are prone to negative emotions, reducing their professional identity and sense of value. Studies have shown that work well-being can also be used as an effective coping mechanism to improve individual cognition, behavior, and ability [55], which enables nurses to re-evaluate and reflect on their careers, actively find their available resources to cope with work, strengthen communication and exchange with patients, implement narrative nursing for patients, and constantly improve individual medical narrative ability in practice.

In conclusion, nursing administrators and educators should pay attention to the important influence of nurses' perceived organizational support and work well-being on medical narrative ability. The organizational support theory [56] can be used to manage the human resources of nurses, formulate reasonable performance appraisal plans, improve the salary subsidies of employees, pay attention to the emotional needs and work pressure of employees, and so on to improve nurses' perceived organizational support and further improve their medical narrative ability. The purpose of Williams Life Skills Training is to relieve the practitioner's self-pressure, psychological burden, and stress [57]. Research shows that Williams life skills training can be used to intervene in nurses' work well-being, relieve their negative emotions [58], and improve nurses' medical narrative ability. In addition, the research pointed out that the medical narrative ability of nurses could be improved by strengthening the theoretical and practical training of narrative medicine or narrative nursing [59], training nurses specialized in narrative nursing, and conducting clinical case analysis [60]. An interesting study pointed out that nurses' creativity can be improved by reading literary works, and they can feel the meaning of birth, old age, illness, death, and life. It is easier to see the nature behind the disease, and their empathy ability can be improved further to affect nurses' medical narrative ability [61]. Such intervention methods have not yet appeared in China and are worth further exploration in the future. Finally, we should also pay attention to the influence of nurses' marital status, working hours and departments, whether they participate in social activities every week and other factors on nurses' medical narrative ability, and formulate targeted intervention measures according to individual conditions, to improve medical narrative ability and optimize nursing quality [62].

### 4.1. Limitations and Future Research

Although this study provides some value for researching the relationship between nurses' medical narrative ability, work well-being, and perceived organizational support, it has several limitations to consider. First, the study used convenience sampling to select participants from eight hospitals in different regions of China, which may limit the generality of the results. It is suggested that stratified random sampling should be considered in future sample selection, which will enhance the universality of the study results across different regions and different types of medical institutions. Second, the nature of cross-sectional studies limits the possibility of drawing causal relationships between variables. To better understand the relationship between nurses' medical narrative ability and work well-being, it is suggested that prospective longitudinal studies could be conducted in the future to explore causality, which would allow researchers to track changes over time and establish a clearer causal dynamic. Finally, the study relied on self-reported data, which may have been driven by response bias or a desire to be presented in a socially desirable way. Future research suggests adding objective indicators, which could include performance evaluations or observational studies to supplement self-reported questionnaires. We can also explore other variables that influence medical narrative ability in the future, which will give us a more comprehensive understanding of the factors that influence medical narrative ability.

## 5. Conclusions

In this cross-sectional multicenter study, we conclude that the perceived organizational support, work well-being, and medical narrative ability of Chinese nurses need to be improved. There is a positive correlation between nurses' perceived organizational support, work well-being, and medical narrative ability, which emphasizes the important mediating role of work well-being between perceived organizational support and medical narrative ability. This study has enriched the theoretical understanding of the relationship between perceived organizational support, work well-being, and medical narrative ability, provided new ideas and practical strategies for medical and educational institutions to develop appropriate interventions to improve nurses' medical narrative ability, and also provided a solid foundation for future research and practical application in this field.

## 6. Implications for Nursing Management

The results of this study provide new insights for nursing managers to take relevant measures to improve nurses' medical narrative ability, considering that the perceived organizational support and work well-being have beneficial effects on nurses' medical narrative ability. Therefore, nurse managers should pay attention to nurses' perceived organizational support, work well-being, and medical narrative ability and provide targeted intervention measures. For example, nursing managers can improve the working environment of nurses by rationally arranging human resources and establishing an effective reward system and title promotion mechanism [63], thus enhancing their perceived organizational support and medical narrative ability. Mindfulness and stress reduction training, meditation practice, and other methods can also be used to intervene in nurses' mental health problems, such as work pressure [64], and improve nurses' work well-being and medical narrative ability. Finally, nursing managers can improve nurses' medical narrative ability by strengthening narrative nursing training, cultivating narrative nursing professionals, and carrying out narrative nursing clinical practice [65] to optimize nursing quality, which is conducive to patients' physical and mental health and promotes humanistic nursing development.

## Figures and Tables

**Table 1:** Comparison of main study variable among nurses with different sociodemographic characteristics (*n* = 1831).

Sociodemographic characteristics	Number (%)	Medical narrative ability (mean ± SD)	Perceived organizational support (mean ± SD)	Work well-being (mean ± SD)
*Gender*				
Female	1783 (97.4)	154.43 ± 22.95	46.73 ± 10.97	53.16 ± 10.77
Male	48 (2.6)	156.13 ± 22.52	44.85 ± 11.86	50.29 ± 12.04
*t*/*F*		−0.505	1.166	1.818
*p*		0.614	0.244	0.069

*Age (years)*				
18–25	210 (11.5)	151.57 ± 19.93	46.95 ± 10.11	51.22 ± 9.56
26–30	601 (32.8)	154.01 ± 23.90	47.15 ± 11.12	52.59 ± 10.76
31–35	500 (27.3)	154.11 ± 23.04	46.03 ± 11.18	53.10 ± 11.02
36–40	259 (14.1)	154.52 ± 21.90	47.14 ± 10.84	52.88 ± 11.09
41–45	124 (6.8)	158.06 ± 21.39	46.98 ± 11.01	54.85 ± 10.44
> 45	137 (7.5)	159.00 ± 25.03	45.43 ± 11.36	56.93 ± 10.96
*t*/*F*		2.433	1.059	5.728
*p*		0.033	0.381	< 0.001

*Marital status*				
Married	1348 (73.6)	154.89 ± 23.33	46.60 ± 11.24	53.77 ± 0.94
Single	410 (22.4)	152.00 ± 21.92	46.88 ± 10.37	50.74 ± 10.21
Divorce	73 (4.0)	160.79 ± 19.40	46.96 ± 9.91	53.77 ± 9.99
*t*/*F*		5.403	0.125	12.589
*p*		0.005	0.882	0.688

*Education level*				
High school	12 (0.7)	163.83 ± 17.45	45.00 ± 12.79	55.00 ± 12.92
College	511 (27.9)	154.94 ± 22.52	46.78 ± 10.52	52.78 ± 10.70
University or above	1308 (71.4)	154.21 ± 23.13	46.66 ± 11.17	53.19 ± 10.84
*t*/*F*		1.190	0.166	0.451
*p*		0.304	0.847	0.637

*Department*				
Internal medicine	688 (37.6)	151.17 ± 23.42	45.86 ± 11.27	51.50 ± 10.65
Surgical	432 (23.6)	155.86 ± 22.95	47.03 ± 11.13	53.47 ± 11.03
Pediatric	86 (4.7)	159.34 ± 19.31	50.23 ± 8.88	56.28 ± 9.17
Obstetrics and gynecology	115 (6.3)	158.16 ± 22.11	47.66 ± 10.37	54.58 ± 11.15
Outpatient	83 (4.5)	162.37 ± 17.29	47.75 ± 10.70	54.10 ± 10.88
Emergency	87 (4.8)	155.10 ± 19.19	45.01 ± 11.32	54.30 ± 10.74
Others	340 (18.6)	154.84 ± 24.14	46.83 ± 10.78	53.95 ± 10.77
*t*/*F*		5.528	2.853	4.894
*p*		< 0.001	0.009	3.66

*Working experience (years)*				
< 3	212 (11.6)	149.91 ± 22.22	47.41 ± 9.72	51.36 ± 9.82
3–5	319 (17.4)	152.88 ± 23.16	47.33 ± 10.85	52.32 ± 10.33
6–10	426 (23.3)	154.33 ± 22.08	46.40 ± 11.00	52.46 ± 10.78
11–15	521 (28.5)	155.63 ± 23.25	46.04 ± 11.58	53.23 ± 11.27
> 15	353 (19.3)	157.14 ± 23.27	46.95 ± 10.97	55.38 ± 10.84
*t*/*F*		4.033	1.073	6.162
*p*		0.003	0.368	< 0.001

*Income (RMB/month)*				
< 3000	106 (5.8)	149.52 ± 22.08	45.71 ± 11.89	49.15 ± 9.60
3000–5000	699 (38.2)	153.95 ± 24.49	46.65 ± 11.65	53.14 ± 11.06
5001–10000	978 (53.4)	155.35 ± 21.79	46.64 ± 10.49	53.23 ± 10.62
> 10,000	48 (2.6)	155.31 ± 23.07	49.96 ± 8.87	58.15 ± 11.10
*t*/*F*		2.268	1.704	8.347
*p*		0.079	0.164	< 0.001

*Receive narrative medicine training*				
No	908 (49.6)	150.11 ± 23.43	46.73 ± 10.97	51.23 ± 10.29
Yes	923 (50.4)	158.77 ± 21.60	44.85 ± 11.86	54.92 ± 11.01
*t*/*F*		−8.219	−6.792	−7.396
*p*		< 0.001	0.689	0.522

*Participate in social activities every week*				
No	757 (41.3)	151.35 ± 24.95	45.11 ± 11.34	51.01 ± 10.71
Yes	1074 (58.7)	156.68 ± 21.13	47.79 ± 10.68	54.56 ± 10.65
*t*/*F*		−4.795	−5.167	−7.002
*p*		< 0.001	< 0.001	0.056

Abbreviation: SD, standard deviation.

**Table 2:** Descriptive analyses of medical narrative ability, work well-being, and perceived organizational support (*n* = 1831).

Variables	Min	Max	Mean ± SD (item mean score)
*Medical narrative ability*			
Pay attention and listen	19	63	50.06 ± 7.41
Understanding and response	12	84	69.48 ± 11.34
Reflection and representation	6	42	34.94 ± 5.66
Total score	38	189	154.48 ± 22.93

*Work well-being*			
Positive emotion	4	20	13.93 ± 3.55
Negative emotion	5	25	18.19 ± 5.36
Work satisfaction	6	30	20.97 ± 4.84
Total score	15	75	53.09 ± 10.81

*Perceived organizational support*			
Emotional support	10	50	35.42 ± 8.70
Instrumental support	3	15	11.26 ± 2.57
Total score	13	65	46.68 ± 11.00

Abbreviations: Max, maximum; Min, minimum; SD, standard deviation.

**Table 3:** Correlation analyses between medical narrative ability, work well-being, and perceived organizational support (*n* = 1831).

Variables	Medical narrative ability	Work well-being	Perceived organizational support
Medical narrative ability	1		
Work well-being	0.391⁣^∗∗^	1	
Perceived organizational support	0.348⁣^∗∗^	0.685⁣^∗∗^	1

⁣^∗∗^*p* < 0.01.

**Table 4:** Mediating effect of work well-being between perceived organizational support and medical narrative ability (*n* = 1831).

Effect	Path	*β*	Bootstrap 95% CI	SE	*t*	*p*
Direct effect	Perceived organizational support ⟶ medical narrative ability	0.320 (c′)	0.200, 0.440	0.061	5.221	< 0.001

Indirect effect	Perceived organizational support ⟶ work well-being	0.657 (a)	0.624, 0.689	0.017	39.725	< 0.001
	Work well-being ⟶ medical narrative ability	0.534 (b)	0.409, 0.658	0.064	8.408	< 0.001

Total effect	Perceived organizational support ⟶ medical narrative ability	0.670 (c)	0.580, 0.760	0.046	14.676	< 0.001

*Note:* Adjusting for covariates, including age, marital status, department, working experience, receive narrative medicine training, and participate in social activities every week.

Abbreviations: CI, confidence interval; SE, standard error.

## Data Availability

The data that support the findings of this study are available from the corresponding author upon reasonable request.
